# Can clinicians predict individual patient outcomes in neuroendocrine tumors treated with [^177^Lu]Lu-DOTATATE?

**DOI:** 10.1093/oncolo/oyag231

**Published:** 2026-06-15

**Authors:** Javier López-Robles, Mercedes Mitjavila, Paula Jimenez-Fonseca, Isabel Marín-Melero, Pilar Belló, Virginia Pubul, Amparo Garcia-Burillo, Jorge Hernando, Belén Llana, Julián Ardila, Javier Arbizu, Rocío Valverde, Mónica Velasco, Maribel Castellón, Teresa Alonso-Gordoa, Lina García-Cañamaque, Juana María Cano, María Josefa Tabuenca, María Carmen Riesco, Ana Belén Custodio, Adrián Piñeiro, David Balaguer-Muñoz, Marina Nevares, Alberto Carmona-Bayonas

**Affiliations:** Medical Oncology Department, Hospital Universitario Morales Meseguer, University de Murcia, IMIB, Murcia, 30008, Spain; Nuclear Medicine Department, Hospital Universitario Puerta de Hierro, Majadahonda, 28222, Spain; Medical Oncology Department, Hospital Universitario Central de Asturias, ISPA, Oviedo, 33011, Spain; Nuclear Medicine Department, Institute Jules Bordet, Brussels, 1070, Belgium; Nuclear Medicine Department, Hospital Universitario La Fe, Valencia, 46026, Spain; Nuclear Medicine Department, Hospital Clínico Universitario de Santiago, Santiago de Compostela, 15706, Spain; Nuclear Medicine Department, Hospital Universitario Vall d‘Hebron, Barcelona, 08035, Spain; Medical Oncology Department, Hospital Universitario Vall d‘Hebron, VHIO, Barcelona, 08035, Spain; Nuclear Medicine Department, Hospital Universitario Central de Asturias, Oviedo, 33011, Spain; Nuclear Medicine Department, Hospital Universitario Gregorio Marañón, Madrid, 28007, Spain; Nuclear Medicine Department, Clínica Universidad de Navarra, Pamplona, 31008, Spain; Nuclear Medicine Department, Hospital Universitario de Cruces, Baracaldo, 48903, Spain; Nuclear Medicine Department, Hospital of la Santa Creu i San Pau, Barcelona, 08041, Spain; Nuclear Medicine Department, Hospital Universitario Virgen de la Arrixaca, Murcia, 30120, Spain; Medical Oncology Department, Hospital Universitario Ramón y Cajal, Madrid, 28034, Spain; Nuclear Medicine Department, Hospital HM Sanchinarro, Madrid, 28050, Spain; Medical Oncology Department, Hospital General Universitario de Ciudad Real, Ciudad Real, 13005, Spain; Nuclear Medicine Department, Hospital Universitario 12 de Octubre, Madrid, 28041, Spain; Medical Oncology Department, Hospital Universitario 12 de Octubre, Madrid, 28041, Spain; Medical Oncology Department, Hospital Universitario La Paz, Madrid, 28046, Spain; Nuclear Medicine Department, Hospital Universitario Virgen de las Nieves, Granada, 18014, Spain; Nuclear Medicine Department, Hospital Universitario Doctor Peset, Valencia, 46017, Spain; Nuclear Medicine Department, Hospital Universitario de Burgos, Burgos, 30008, Spain; Medical Oncology Department, Hospital Universitario Morales Meseguer, University de Murcia, IMIB, Murcia, 30008, Spain

**Keywords:** neuroendocrine tumors, nomogram, peptide receptor radionuclide therapy, prognosis, progression-free survival, [^177^Lu]Lu-DOTATATE

## Abstract

**Background:**

Peptide receptor radionuclide therapy (PRRT) has become a cornerstone in the management of neuroendocrine tumors (NETs), yet optimal sequencing and patient selection remain unsettled. This study aimed to develop and internally validate the NEPTUNE score to predict progression-free survival (PFS) in advanced NETs patients receiving [^177^Lu]Lu-DOTATATE.

**Materials and Methods:**

Real-world data from the nationwide SEPTRALU registry and the Jules Bordet Institute included patients with advanced NETs treated with [^177^Lu]Lu-DOTATATE. Predictors of PFS were identified using an accelerated failure time model and combined into the NEPTUNE score. Internal validation was performed using bootstrap resampling. This tool was subsequently transformed into a nomogram and an interactive web-based calculator to enhance its integration into routine clinical practice.

**Results:**

The cohort comprised 647 patients with diverse NET subtypes: pancreatic (39%), midgut (30%), bronchopulmonary (9%), pheochromocytoma/paraganglioma (3%), other gastroenteropancreatic (11%), and other non-gastroenteropancreatic (8%). The NEPTUNE score incorporates ten routinely available variables: ECOG performance status, PRRT line, Ki67 index, number of metastatic sites, primary tumor site, sex, Krenning grade, surgical resection of metastases, presence of liver metastases, and time from advanced tumor diagnosis to PRRT. The score demonstrated strong performance, with an Integrated Brier Score of 0.201 and a bias-corrected C-index of 0.702, indicating good calibration and discrimination.

**Conclusions:**

The NEPTUNE score is a promising tool for predicting individual PFS in patients with advanced NETs treated with [^177^Lu]Lu-DOTATATE. By integrating readily available clinical variables, it may support clinical decision-making. However, external validation is required before broader clinical implementation.

## Graphical abstract

**Figure:**
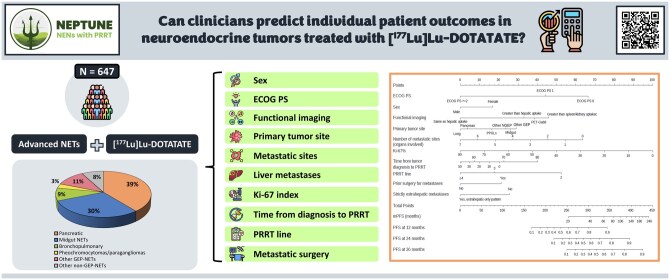

Implications for Practice The NEPTUNE model enables practical, individualized estimation of progression-free survival in patients with neuroendocrine tumors considered for [^177^Lu]Lu-DOTATATE therapy. By integrating routinely available clinical variables, performance status, Ki67 index, tumor burden and distribution, primary site, functional imaging uptake, prior metastatic surgery, treatment line, sex, and timing of PRRT, the model can be applied at the point of care to support patient selection, optimize treatment sequencing, and guide multidisciplinary discussions. Its use may improve alignment between expected benefit and therapeutic intent, facilitating more informed clinical decision-making and patient counseling.

## Introduction

Neuroendocrine tumors (NETs) comprise a heterogeneous group of tumors originating from neuroendocrine cells, which are widely distributed throughout the body, including the gastrointestinal tract, lungs, and various endocrine glands.^1^^,^^2^ Once considered rare, the incidence of NETs has risen substantially in recent years, posing an increasingly significant clinical challenge.^3^^,^^4^ This challenge is largely attributed to the marked variability in NETs’ biological behavior, resulting in a wide spectrum of prognoses and clinical outcomes, as evidenced by large-scale population-based studies. For example, data from the Surveillance, Epidemiology, and End Results registry report a median overall survival (OS) of 9.3 years for NETs, with substantial variation according to primary tumor site and tumor grade.^2^

Notwithstanding their substantial clinical and pathological heterogeneity, a common molecular feature frequently observed in NETs is the expression of somatostatin receptors (SSTRs). This is particularly notable in gastroenteropancreatic (GEP) NETs and bronchopulmonary (BP) NETs, where SSTRs are present in up to 80% and 50% of cases, respectively.^3^^,^^5^ The prevalence of this shared molecular target has spurred the development and application of somatostatin analogs (SSAs) and peptide receptor radionuclide therapy (PRRT) across a diverse range of histological subtypes.^3^^,^^5^^,^^6^ Consequently, upon progression during SSA therapy, [177Lu]Lu-DOTATATE often emerges as the most promising therapeutic strategy in various clinical scenarios.^6^ The landmark phase III NETTER-1 trial provided pivotal evidence supporting this approach, demonstrating that [177Lu]Lu-DOTATATE significantly improved outcomes compared to high-dose octreotide in patients with advanced midgut NETs progressing on standard SSA therapy.^7^ More recently, the phase III NETTER-2 study further reinforced the efficacy of [177Lu]Lu-DOTATATE, revealing improved response rates and progression-free survival (PFS) in newly diagnosed, advanced GEP-NETs with a Ki67 index ranging from 10% to 55%.^8^ These findings support broader use of [177Lu]Lu-DOTATATE across SSTR-expressing subtypes, making it essential to identify factors predictive of poorer outcomes.^9^

Predicting PFS in NETs is a complex challenge. While numerous prognostic factors have been identified, including functional status, tumor biology, prior treatments, biomarkers, and imaging findings,^3^ relying on any single factor fails to capture the intricate interplay that ultimately determines patient outcomes. This complexity underscores the need for more sophisticated, integrative prognostic approaches.

To date, only a handful of multivariable prognostic models have been developed for NET patients undergoing PRRT. These “first-generation” models often lack crucial parameters like Ki67 proliferation index or treatment line.^10^ To address this gap, we developed and internally validated the NEPTUNE score, which incorporates critical variables, including prior cytoreduction and detailed tumor burden, to provide clinicians with a more accurate tool for predicting PFS in advanced NETs receiving PRRT.

## Materials and methods

### Study design and population

The Spanish Society of Nuclear Medicine and Molecular Imaging (SEMNIM) established SEPTRALU (NCT04949282), a nationwide registry designed to prospectively collect data on patients with NETs undergoing PRRT. The registry includes adult patients diagnosed with histologically confirmed, unresectable and/or metastatic NETs that express SSTRs, regardless of tumor grade or primary site; patients with poorly differentiated neuroendocrine carcinomas were not included. To date, 26 centers across Spain and one center in Belgium (the Jules Bordet Institute) are actively contributing patient data. All participants enrolled in the SEPTRALU registry received [177Lu]Lu-DOTATATE administered intravenously at a dose of 7.4 GBq every 8 weeks as standard therapy. Follow-up data were collected for a minimum of 3 months, unless a patient expired earlier. A dedicated online platform with integrated validation checks ensures data integrity and enables real-time, web-based monitoring of case data quality.^9^

The study was approved by the Research Ethics Committee of the Principality of Asturias (151/17, November 3, 2017) and by the Spanish Agency of Medicines and Medical Devices as a post-authorization study with prospective follow-up (CSV: DSRZJ6QF1B). Procedures complied with institutional, local, and national standards and the 1964 Helsinki Declaration and later amendments. Informed consent was obtained from all patients before being included in the study.

### Variables of interest

The primary endpoint of this study was PFS, defined as the time elapsed from the initiation of [^177^Lu]Lu-DOTATATE therapy to either tumor progression or death from any cause. Patients lost to follow-up were censored at their last known follow-up date. An extensive set of potential predictor variables was initially considered, derived from a comprehensive literature review and expert consensus. This initial pool encompassed variables spanning demographic characteristics, clinical parameters, laboratory values, functional imaging findings, tumor-specific attributes, and prior treatment history. From this comprehensive set, 10 variables were ultimately selected based on their established relevance in the literature and expert opinion: the number of metastatic sites, Ki67 proliferation index, time from diagnosis of advanced disease to initiation of PRRT, surgical resection of metastases, presence of extrahepatic-only metastases, ECOG performance status (PS), sex, functional imaging findings, primary tumor site, and PRRT line.

Functional imaging findings were harmonized across modalities and incorporated into the model as an ordinal variable reflecting increasing somatostatin receptor expression; full definitions and mapping are provided in Table S1.

### Statistical analyses

To address covariate redundancy, we evaluated multicollinearity using variance inflation factors, pairwise correlations, redundancy analysis, and hierarchical clustering with the Hoeffding D statistic,^11^ which did not identify significant redundancy or dependency among covariates. To account for potential heterogeneity across tumor subtypes, we explored interactions between primary tumor site and key predictors, including the Ki-67 proliferation index and PRRT line, as well as interactions involving tumor grade; these terms did not improve model performance and were not retained.

PFS was modeled using an accelerated failure time (AFT) model with a Weibull distribution, chosen for its smooth PFS estimates (vs Cox’s stepwise outputs) and for its suitability for indolent tumors, where prognostic factors evolve over time.^12–16^ In the AFT model, survival times are accelerated or decelerated by yielding time ratios (TRs): TRs <1 indicate a faster event rate, while TRs >1 suggest a delay. For binary predictors, log(0.5) represents a halving of the median event time. Nonlinear relationships were modeled with restricted cubic splines, guided by the Akaike Information Criterion. Candidate variables with more than 20% missing data (eg, chromogranin A, symptom burden) were excluded a priori, in line with the recommendation that multiple imputation becomes unreliable beyond this threshold and may introduce bias that outweighs the information recovered. The missingness profile of all candidate predictors is provided in Table S2. Variables with less than 20% missingness were imputed using predictive mean matching with the aregImpute function (Hmisc package), generating 10 multiply imputed datasets; the imputation model included all candidate predictors together with the outcome (time and event indicator) and baseline covariates (age, year of treatment). Parameter estimates were pooled across imputations using Rubin’s rules. A sensitivity analysis comparing the multiple-imputation and complete-case fits (*n* = 560, 86.6%) showed closely concordant coefficients, discrimination, and calibration (Table S3). The fixed sample size (∼15 events per predictor) ensured model feasibility.

Performance was assessed with calibration graphs, Harrell’s C-index (bias-corrected via 1000 bootstraps), and an experimental “interval-censored C-index,” which considers patients with similar predicted risk as concordant if PFS differ by less than 3 months, a threshold based on typical imaging intervals in clinical trials.^17^ This modified C-index reduces penalties for minor PFS differences, focusing on clinically meaningful variations. This metric was considered exploratory and was intended to complement, rather than replace, the primary performance measures (see R code below). Additionally, the Integrated Brier Score (IBS) was used to evaluate overall prediction accuracy over time. The same model specifications and performance metrics were applied to predict OS. All analyses were performed in R version 3.5.1, using the SurvMetrics, Hmisc, and rms packages. The dataset and R code used in this study are available at Figshare.^18–21^

## Results

### Baseline characteristics

The final dataset included 647 patients. Their baseline characteristics are summarized in Table 1. The cohort was predominantly male (59%), with a median age of 62 years (range: 18-88 years). Most patients (95%) had a good performance status (ECOG PS 0-1). The most common primary tumor site was the pancreas (pancreatic NETs [pNETs]; 39%), followed by midgut NETs (30%), other gastroenteropancreatic NETs (GEP-NETs; 11%), bronchopulmonary NETs (BP-NETs; 9%), other non-GEP-NETs (8%), and pheochromocytomas and paragangliomas (PPGLs; 3%). The majority of tumors were nonfunctioning (71%). The median Ki67 proliferation index was 5% (range: 1-80). Functional imaging was performed using ^68^Ga-DOTATOC PET in one-third of cases and with somatostatin receptor scintigraphy in the remaining two-thirds. Of those staged with scintigraphy, a Krenning grade of 3, indicating uptake greater than hepatic uptake, was the most frequent pattern, observed in 73% of cases. Regarding prior treatment, 38% of patients received PRRT as a second-line therapy. The median time from oncological diagnosis to the initiation of PRRT was 40 months (range: 2-287 months). The most common prior treatments included SSAs in 95% of cases, everolimus in 42%, chemotherapy in 24%, and sunitinib in 17% (Supplementary Appendix).

**Table 1.** Baseline characteristics.

Baseline characteristics	*N* (%)
**Age (years) – median (range)**	62 (18-88)
**Men**	382 (59)
**Women**	265 (41)
**ECOG PS**	
** 0**	337 (52)
** 1**	279(43)
** ≥2**	31 (5)
**Ki67% – median (range)**	5 (1-80)
**Primary tumor site**	
** Pancreatic**	253 (39)
** Midgut**	194 (30)
** Other GEP**	71 (11)
** Bronchopulmonary**	59 (9)
** Other non-GEP**	50 (8)
** Pheochromocytomas/paragangliomas**	20 (3)
**WHO 2019**	
** NET G1**	209 (32)
** NET G2**	374 (58)
** NET G3**	64 (10)
**Hormonal syndrome**	186 (29)
**Localization of metastases**	
** Liver**	562 (87)
** Peritoneum**	103 (16)
** Lymph nodes**	365 (56)
** Lung**	52 (8)
** Bone**	159 (25)
**Functional imaging assessment**	
** ^68^Ga-DOTATOC-PET**	188 (29)
** Scintigraphy—Krenning G2**	22 (3)
** Scintigraphy—Krenning G3**	336 (52)
** Scintigraphy—Krenning G4**	101 (16)
**Prior surgery**	
** Primary tumor**	352 (54)
** Metastases**	153 (24)
**Prior locoregional and ablative therapies**	90 (14)
**Number of prior systemic treatments**	
** 1**	244 (38)
** 2**	213 (33)
** ≥3**	190 (29)
**Prior systemic treatments**	
** Somatostin analogues**	612 (95)
** Sunitinib**	112 (17)
** Everolimus**	272 (42)
** Chemotherapy**	157 (24)
**Time (months) from diagnosis to PRRT—median (range)**	40 (2-287)
**Total**	647 (100)

Abbreviations: ECOG PS, Eastern Cooperative Oncology Group Performance Status; G, Grade; GEP, gastroenteropancretic; NET, neuroendocrine tumor; PET, positron emission tomography; PRRT, peptide receptor radionuclide therapy; WHO: World Health Organization.

### Efficacy and survival-based endpoints

**Figure 1.**
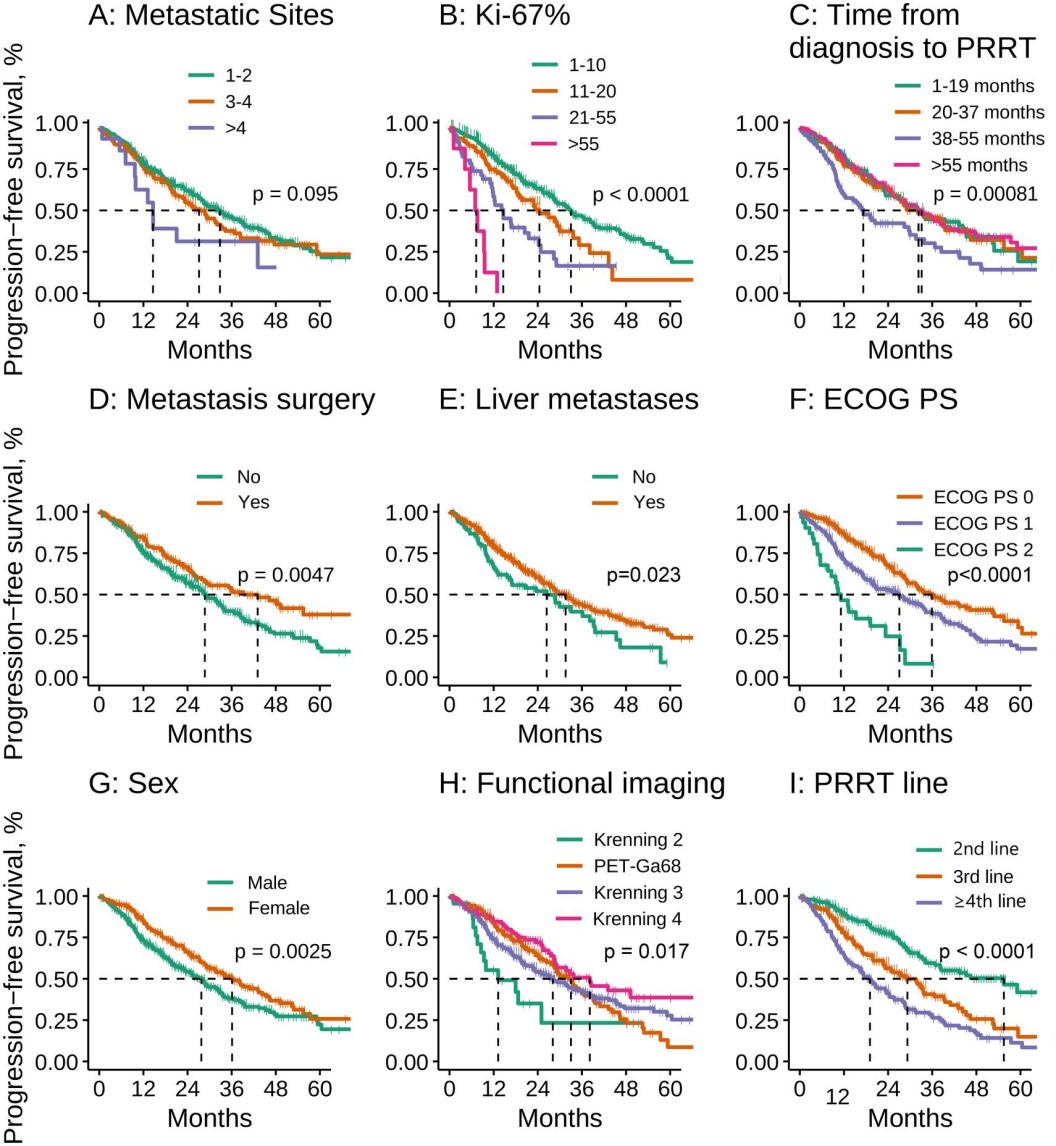
PFS curves stratified by key clinical and prognostic variables. PFS curves are displayed for stratifications based on key clinical and biological covariates, with log-rank test *P*-values provided for each comparison. Abbreviations: ECOG PS, Eastern Cooperative Oncology Group Performance Status; PET, positron emission tomography; PFS, progression-free survival; PRRT, peptide receptor radionuclide therapy.

After a median follow-up of 29 months for patients alive at the time of analysis, 305 progression events and 183 deaths were recorded, including 3 deaths without documented progression. The median PFS was 29.9 months (95% CI: 27.6-33.8 months), and the median OS was 49.6 months (95% CI: 44.8-59.3 months). Notably, survival outcomes varied considerably by tumor subtype. Patients with midgut tumors exhibited a median PFS of 33.0 months (95% CI: 28.5-46.4 months), whereas those with pancreatic tumors or other subtypes had a median PFS of 26.4 months (95% CI: 22.8-31.8 months). Figure 1 illustrates the survival outcomes stratified by key clinical and prognostic variables, while detailed response breakdowns are provided in the Supplementary Appendix.

### Development of the NEPTUNE predictive model for PFS

The AFT model incorporated 10 key covariates to comprehensively capture the prognosis of patients with NETs. These covariates, ordered by their decreasing association with time to PFS as measured by Somer’s *D* (detailed methodology in the Supplementary Appendix), were ECOG PS, PRRT line, Ki67 proliferation index, number of metastatic sites, primary tumor site, sex, Krenning grade, surgical resection of metastases, presence of liver metastases, and time from diagnosis of advanced disease to initiation of PRRT.

**Figure 2.**
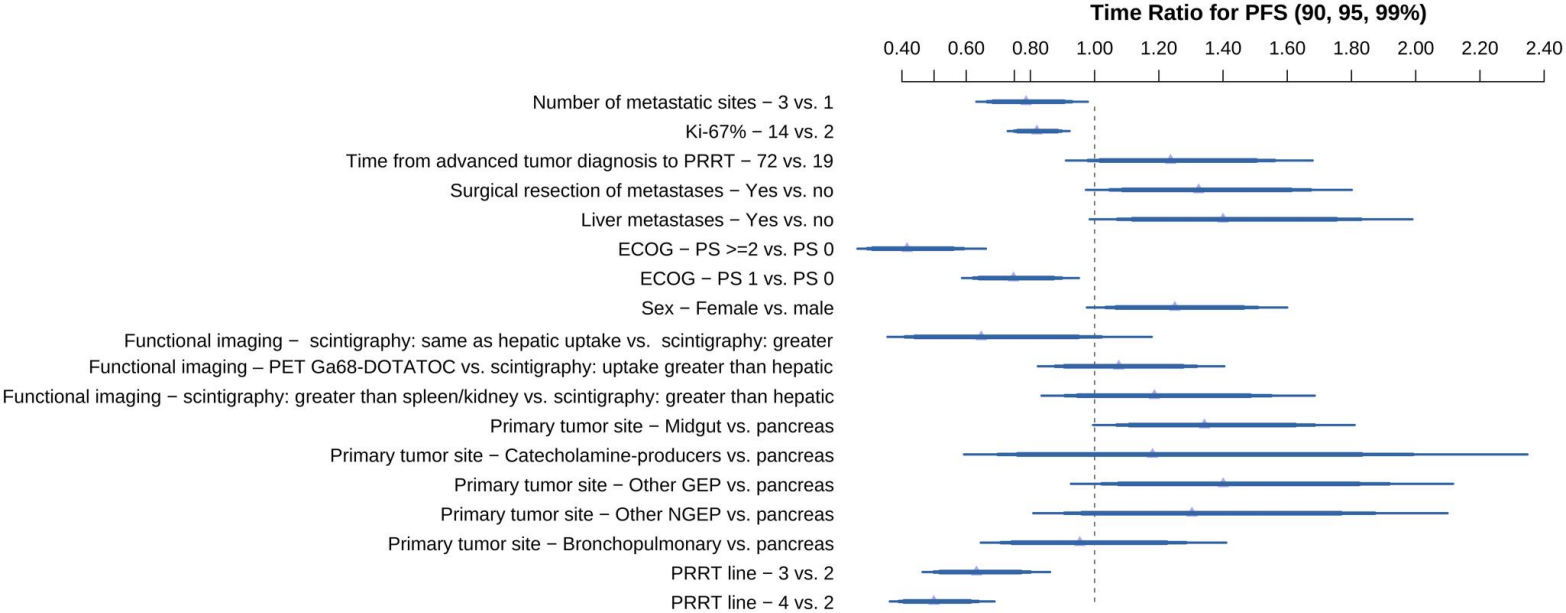
AFT model time ratios for PFS. In an AFT model, the time ratio indicates how the expected event time is accelerated or decelerated for one group relative to another. For example, a time ratio of 2 doubles the median survival time (improved prognosis), while 0.5 halves it (worse prognosis). Abbreviations: AFT, accelerated failure time; ECOG PS, Eastern Cooperative Oncology Group Performance Status; GEP, gastroenteropancreatic; NGEP, non-gastroenteropancreatic; PET, positron emission tomography; PFS, progression-free survival; PRRT, peptide receptor radionuclide therapy.

The AFT model identified ECOG PS, Ki67 proliferation index, and PRRT line as the most influential predictors of PFS. Tumor burden, sex, presence of liver metastases, and surgical resection of metastases also contributed significantly to the model. Furthermore, the time from diagnosis of advanced disease to initiation of PRRT, modeled using a non-linear approach, provided additional prognostic information. In this dataset, the linearity assumption was valid for both the Ki67 proliferation index and the number of metastatic sites. Detailed survival time ratios and confidence intervals are provided in Table 2 and visually represented in Figure 2.

**Table 2.** Survival time ratios with confidence intervals for PFS.

Variables	Contrast	**Time ratio (95% CI)** ^a^
**Number of metastatic sites**	High: 3, Low: 1	0.78 (0.66-0.92)
**Ki67 index**	High: 14, Low: 2	0.82 (0.74-0.89)
**Time from advanced tumor diagnosis to PRRT (months)**	High: 72, Low: 19	1.23 (0.97-1.56)
**Surgical resection of metastases**	Yes vs No	1.32 (1.04-1.67)
**Strictly extrahepatic metastases**	No vs Yes	1.39 (1.07-1.83)
**ECOG PS**	≥2 vs 0	0.41 (0.29-0.59)
	1 vs 0	0.74 (0.62-0.89)
**Sex**	Female vs Male	1.24 (1.03-1.50)
**Functional imaging**	Same as Hepatic Uptake vs Greater	0.64 (0.40-1.02)
	PET vs Greater than Hepatic Uptake	1.07 (0.87-1.31)
	Greater than Spleen/Kidney vs Greater than Hepatic	1.18 (0.90-1.55)
**Primary tumor site**	Midgut vs Pancreas	1.34 (1.06-1.68)
	Catecholamine-Producers vs Pancreas	1.18 (0.69-1.99)
	Other GEP vs Pancreas	1.40 (1.02-1.91)
	Other non-GEP vs Pancreas	1.30 (0.90-1.87)
	Lung vs Pancreas	0.95 (0.70-1.28)
**PRRT line**	3rd vs 2nd	0.63 (0.49-0.80)
	4th vs 2nd	0.49 (0.39-0.63)

a The time ratio quantifies the relative difference between 2 survival times (survival time 1/survival time 2). For instance, patients with an ECOG PS ≥2 experienced a reduced PFS, reflected by a time ratio of 0.41 when compared with those with an ECOG PS of 0. Abbreviations: ECOG PS, Eastern Cooperative Oncology Group Performance Status; GEP, gastroenteropancreatic; PET, positron emission tomography; PFS, progression-free survival; PRRT, peptide receptor radionuclide therapy.

**Figure 3.**
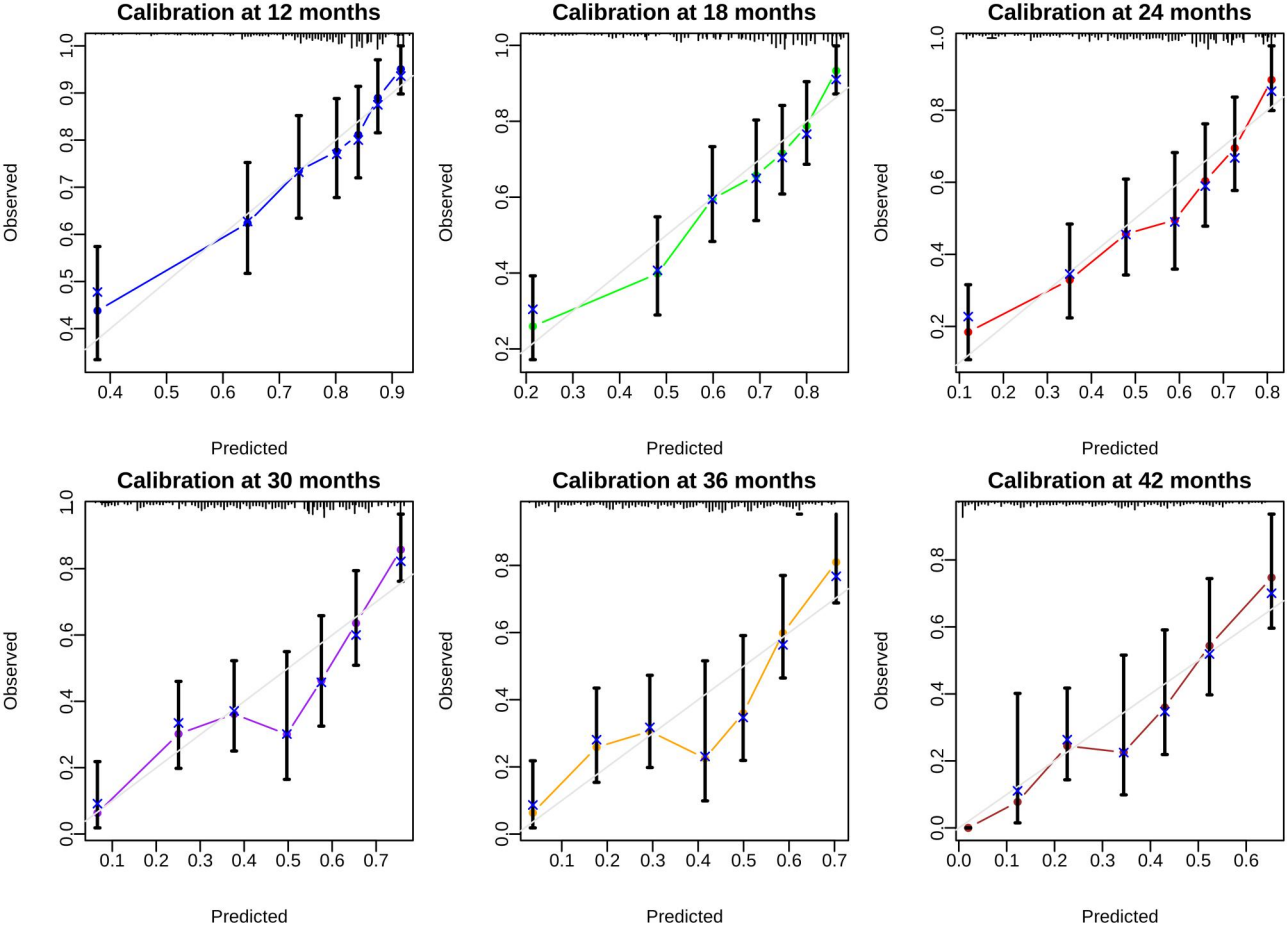
Calibration plots for predicted vs observed PFS rates. Calibration plots compare predicted PFS probabilities at 12, 18, 24, 30, 36, and 42 months with observed progression-free fractions. The 45° line represents perfect prediction; deviations indicate the model’s calibration accuracy. Abbreviation: PFS, progression-free survival.

The model demonstrated good calibration (Figure 3). The IBS was 0.201, indicating good overall predictive accuracy, which is further illustrated for different tumor types in the Supplementary Appendix. The model exhibited a C-index of 0.720 and a bootstrap-corrected C-index of 0.702 (95% CI: 0.670-0.733), signifying good discriminative ability. Harrell’s C-index and the IBS are reported as the primary discrimination and overall accuracy metrics. As a complementary, exploratory analysis, we additionally computed an interval-censored C-index (bootstrap-corrected: 0.735; 95% CI, 0.707-0.772), which is less penalized by minor differences in predicted PFS because progression in routine practice is ascertained at periodic imaging visits rather than continuously; this metric is intended to be interpreted alongside, not in place of, the standard C-index.

**Figure 4.**
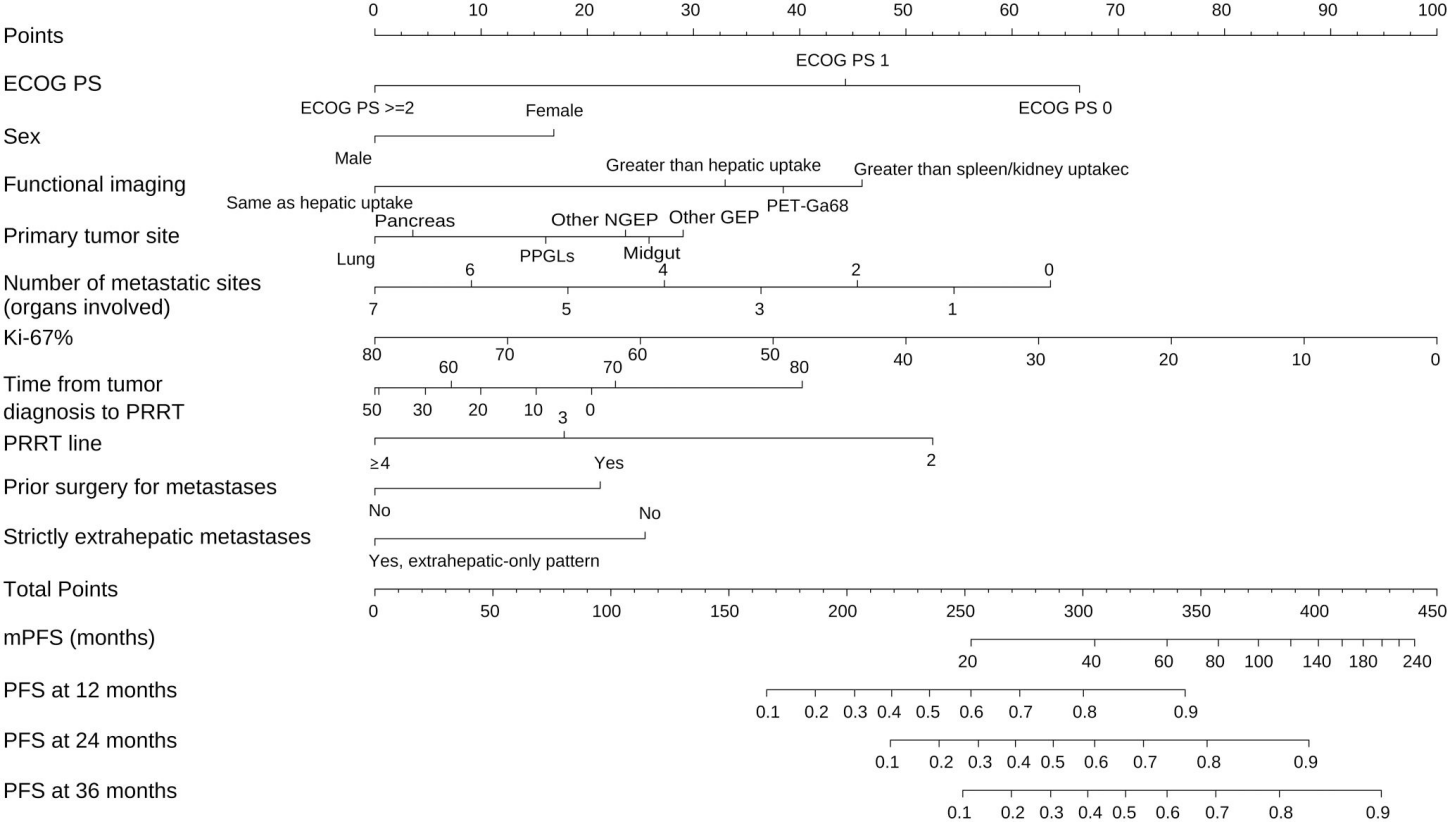
Nomogram for predicting PFS. This nomogram estimates the probability of PFS at 12, 24, and 36 months based on clinical factors. Users align each variable with its corresponding points, sum them, and use the total to predict median PFS and specific PFS rates. Available via an interactive web tool at: https://www.prognostictools.es/clinical-calculators/neptune. Abbreviations: ECOG PS, Eastern Cooperative Oncology Group Performance Status; GEP, gastroenteropancreatic; NGEP, non-gastroenteropancreatic; PET, positron emission tomography; PFS, progression-free survival; PPGLs, paragangliomas/pheochromocytoma; PRRT, peptide receptor radionuclide therapy.

The NEPTUNE for PFS model is presented graphically as a nomogram in Figure 4. For practical clinical application, an interactive web-based tool is available: https://www.prognostictools.es/clinical-calculators/neptune.

The model’s performance using the OS endpoint was assessed by fitting the identical specification and parameterization to these data. The models for PFS and OS, both with the same specification and parameterization, are fully described in the Supplementary Appendix. For the OS endpoint, the model demonstrates a similarly robust performance with a bias-corrected C-index of 0.720 (95% CI, 0.679-0.765). The OS model is also well calibrated across all time points (see Supplementary Appendix).

An illustrative case demonstrating the use of the web-based calculator is provided in Figure S1.

## Discussion

This study introduces and internally validates the NEPTUNE score, a novel predictive tool designed to assist clinicians in estimating prognosis and tailoring treatment strategies for patients with diverse NET subtypes treated with [^177^Lu]Lu-DOTATATE. By integrating readily accessible clinicopathological variables into an easy-to-use score, NEPTUNE facilitates more informed patient selection and personalized treatment planning.

The NEPTUNE score incorporates ten easily obtainable factors, encompassing patient clinical characteristics (ECOG performance status, sex), tumor-specific features (Ki67 proliferation index, number of metastatic sites, primary tumor location, presence of liver metastases), functional imaging findings (Krenning score), and treatment-related aspects (time from diagnosis to PRRT initiation, prior surgical resection of metastases, and PRRT treatment line). This carefully curated combination of variables captures essential elements of patient health status, tumor biology, and treatment history, providing a comprehensive framework for predicting PFS. While these variables are readily available in routine clinical practice, they also possess a sound biological rationale in the context of [^177^Lu]Lu-DOTATATE treatment response, as supported by prior research, thus confirming their relevance for modeling disease progression in patients receiving this therapy.^22–25^

While the primary objective of the NEPTUNE score is to predict PFS based on a comprehensive patient profile, it is worthwhile to consider the impact of individual covariates. Notably, NEPTUNE identifies sex as an independent prognostic factor, with women showing a 24% longer PFS than men (TR, 1.24; *P* = .020), in line with prior reports of poorer outcomes in male NET patients.^26^^,^^27^ Proposed explanations include sex specific tumor sink effects in hepatic and splenic tissues, the influence of gender, diabetes, and age on [^68^Ga]Ga-DOTATOC uptake,^28^ and a possible role of estrogen in GEP-NET biology.^29^ Overall, these proposed mechanisms are hypothetical, and the sex related finding should therefore be interpreted with caution pending external confirmation.

Secondly, the score identified a significant association between prior surgical resection of metastases and improved PFS, with a 32% increase in median PFS. This finding is largely consistent with the existing literature, which supports that [177Lu]Lu-DOTATATE is more effective following surgical debulking.^30^^,^^31^ However, this association should not be interpreted as causal. It is likely influenced, at least in part, by selection factors, as patients eligible for metastasis-directed surgery typically have more favorable disease characteristics, lower tumor burden, or less aggressive tumor biology. Therefore, the observed effect may reflect underlying differences in patient and disease profiles rather than a direct therapeutic impact of surgery itself.

Thirdly, the NEPTUNE score effectively captures the impact of tumor burden on PFS through 2 distinct parameters: the number of metastatic sites and the presence of exclusively extrahepatic disease. Previous studies have established that extrahepatic involvement, particularly in sites such as bone or the central nervous system (CNS), is associated with poorer outcomes in patients with NETs.^32^^,^^33^ While an increased number of both hepatic and extrahepatic metastatic sites reflects a greater overall tumor burden and wider dissemination, the presence of extrahepatic-only disease suggests a distinct and more aggressive biological phenotype, rather than simply a higher disease volume. The NEPTUNE model supports this interpretation: A transition from single-organ to 3-organ metastatic involvement was associated with a median PFS reduction of approximately 22%, and exclusively extrahepatic disease was associated with a further 29% reduction.

Fourthly, the score underscores the crucial influence of timing on the effectiveness of [^177^Lu]Lu-DOTATATE, demonstrating significantly improved outcomes when this therapy is administered earlier in the treatment sequence.^24^^,^^34^ By incorporating the time from diagnosis to PRRT initiation as a covariate, the model reveals a significant and nonlinear correlation with PFS. Specifically, delaying PRRT beyond the initial few years following diagnosis appears to be detrimental, supporting the strategy of earlier PRRT commencement. However, in cases of more indolent tumors, postponing PRRT beyond 3-4 years may offer a seemingly protective effect, suggesting that the favorable tumor biology in these cases might justify a delayed approach. From a molecular perspective, the initial detrimental effect of postponing [^177^Lu]Lu-DOTATATE therapy is likely linked to the gradual loss of SSTR expression. As tumor cells dedifferentiate, their SSTR density may diminish, thereby reducing the efficacy of receptor-targeted therapies. Consequently, earlier treatment may capitalize on higher SSTR expression levels, maximizing therapeutic benefits before substantial biological shifts occur. This hypothesis aligns with observations suggesting that alternative treatment options, such as chemotherapy or targeted therapy, tend to exhibit reduced effectiveness following progression on somatostatin analogs (SSAs).^34^^,^^35^ While this hypothesis appears plausible, its definitive confirmation awaits the results of ongoing clinical trials focused on optimizing therapy sequencing, such as COMPETE (NCT03049189) and COMPOSE (NCT04919226).

Regarding the comparison with other prognostic models, previous studies have investigated factors influencing PFS in patients with NETs treated with PRRT.^36^^,^^37^ However, many of these studies were limited by their reliance on simplistic bivariate associations or a narrow focus on identifying specific causal mechanisms underlying variable patient responses.^36^^,^^37^ In contrast, the development of the NEPTUNE score was driven by the need for a more comprehensive, multivariable approach capable of capturing the complex interplay of multiple factors that collectively shape patient outcomes. Das et al.^36^ introduced a clinical prognostic score derived from a broad NET cohort, comprising 55% midgut and 23% pancreatic NETs. The patient population in our registry-based study differs substantially, with 28.4% midgut and 39% pancreatic tumors. The model by Das et al.^36^ incorporated symptom severity, critical organ involvement (heart, CNS, liver tumor burden >50%), tumor location, presence of peritoneal carcinomatosis, and treatment history into a composite score, where each 1-point increment was associated with a doubling of the hazard for PFS (HR = 2.0; 95% CI: 1.61-2.48). While the score developed by Das et al.^36^ provided valuable insights, it was primarily tailored to midgut and pancreatic NETs. In contrast, the NEPTUNE score accommodates all NET origins, increasing its generalizability across diverse NET subtypes. This broad representation of NET subtypes constitutes a key strength of the model, as it reflects real-world clinical heterogeneity. However, given the well-recognized biological and clinical differences across primary sites and grades, it is possible that site- or grade-specific models could achieve different, and potentially improved, predictive performance within more homogeneous subgroups. Nevertheless, such an approach is limited by the rarity of individual NET subgroups, which would preclude the derivation of stable, adequately powered nomograms for each histology–grade combination; a unified multivariable model that captures heterogeneity through covariates such as primary site, Ki-67, tumor burden, prior surgery and time to PRRT is therefore a pragmatic compromise. Moreover, [^177^Lu]Lu-DOTATATE has been shown to exert activity across NET sites in a largely histology-agnostic manner, provided SSTR expression is preserved, supporting the rationale for a shared prognostic framework.^9^ Other variables were accounted for implicitly. Although tumor grade is a well-established prognostic factor in neuroendocrine neoplasms, the Ki-67 index provides a more precise estimate of biological behavior by virtue of its continuous nature.^3^ For example, a grade 2 tumor with a Ki-67 index of 3% is biologically and clinically distinct from one with a Ki-67 of 20%, despite sharing the same categorical classification. In our model, modeling Ki-67 as a continuous variable with splines subsumes the prognostic information contained in WHO grade; consistent with this, grade did not add independent predictive value in sensitivity analyses and was therefore not retained in the final model.^20^ We did not incorporate symptom burden in the NEPTUNE score due to limitations in documentation; however, we acknowledge its importance, as prior analyses have underscored its likely prognostic value.^3^

This leads us directly to the question of whether we are approaching a ceiling in the predictive accuracy achievable with models based solely on clinical variables. In particular, could we enhance predictive accuracy by incorporating advanced techniques such as radiomics or molecular variables like circulating biomarkers? Some investigators have pursued this avenue, integrating circulating biomarkers into prognostic models for patients undergoing PRRT. For example, Chen et al.^37^ identified elevated baseline chromogranin A levels, normal creatinine levels, and prior chemotherapy exposure as key predictors of post-PRRT progression in a UK-based cohort. These findings underscore the value of combining tumor-specific and systemic biomarkers to achieve a more holistic risk assessment and refine treatment strategies. Similarly, Bodei et al.^38^^,^^39^ have developed 2 innovative blood-based gene signatures: the PRRT prediction quotient, which estimates tumor radiosensitivity, and the NETest, which dynamically correlates with treatment response. Integrating these cutting-edge molecular tools with established clinical parameters, as exemplified by the NEPTUNE score, holds the potential to yield a more robust, refined, and nuanced framework for predicting patient outcomes and guiding personalized treatment decisions in the era of precision medicine.

Regarding the potential contribution of molecular imaging techniques, our pragmatic perspective is to recommend a 2-tiered evaluation, integrating both clinical and functional imaging assessments. The NEPTUNE score demonstrates consistently strong performance and maintains robust calibration across all evaluated time points, indicating a close concordance between predicted and observed PFS. However, incorporating molecular imaging parameters, such as standardized uptake values (SUVs) on [^68^Ga]Ga-DOTATOC PET, tumor heterogeneity, total lesion activity (TLA)/total tumor volume (TV), or lesion detection on [^18^F]F-FDG PET, could further enhance the model’s accuracy. Integrating such parameters, however, would necessitate careful consideration of accessibility, complexity, and cost-effectiveness, as obtaining these additional imaging data typically follows the initial referral to a specialized nuclear medicine center. Therefore, a 2-step approach may offer a practical solution: initial risk stratification using the NEPTUNE score, based on readily available clinical data by endocrinologists and oncologists, could guide the decision to pursue advanced molecular imaging. Subsequently, the model’s predictions could be refined using the richer, more granular insights afforded by PET-based assessments, potentially leading to more personalized treatment strategies.

Several limitations should be acknowledged when interpreting these findings. Firstly, the retrospective nature of the study design inherently carries the risk of introducing inaccuracies and omissions in the collected data. Certain analytical and functional variables, such as chromogranin A, had to be excluded due to insufficient data availability. To mitigate these potential issues, investigators were instructed to meticulously verify data quality in cases where discrepancies or inconsistencies were suspected. Secondly, the relatively short median follow-up period of 29 months may limit the generalizability of the survival analysis, particularly for well-differentiated NETs, which often exhibit an indolent clinical course. The limited number of events observed during the follow-up period increases the uncertainty in the tails of the survival curves. Thirdly, the use of investigator-assessed PFS, while reflective of real-world clinical practice, may not achieve the same level of precision as a centrally adjudicated endpoint. However, it does capture the clinical judgment of treating physicians regarding meaningful changes in disease status that warrant therapeutic intervention. Fourthly, the limited data on first-line treatment within the SEPTRALU registry, which is a reflection of real-world practice patterns, precludes a direct comparison with the aggregated results of the NETTER-2 trial from a first-line perspective.^8^ Furthermore, although the NEPTUNE score demonstrated strong performance after internal validation using bootstrap resampling, its generalizability to independent populations remains uncertain; therefore, external validation in independent cohorts is required before broader clinical implementation. Another limitation of the model is the relative instability of the primary-site coefficients, which showed some variability between the multiple-imputation and complete-case analyses, likely due to reduced stratum sizes. Nevertheless, the direction of these estimates appears clinically plausible, reflecting the generally better prognosis of midgut tumors compared with pancreatic or pulmonary primaries. Finally, the intrinsic heterogeneity of the study population warrants consideration. While the inclusion of a broad spectrum of neuroendocrine tumor subtypes represents a strength by enhancing the applicability of the model to routine clinical practice, differences in tumor biology, clinical behavior, and treatment patterns across primary sites and grades may not be fully captured by a single unified model.

## Conclusion

This study introduces the NEPTUNE score, a novel and concise instrument comprising ten readily available clinical variables, designed to predict PFS in patients with NETs undergoing [^177^Lu]Lu-DOTATATE therapy. The NEPTUNE score represents a promising approach to support personalized treatment decision-making and illustrates the feasibility of estimating PFS using a streamlined set of clinical parameters. In this context, it may serve as an initial assessment tool for clinicians, contributing to more informed treatment planning. It should be noted, however, that the model has undergone internal validation only, and external validation will be necessary to confirm its generalizability.

## Supplementary Material

oyag231_Supplementary_Data

## Data Availability

All the data generated or analyzed in this study are included in the manuscript, tables, and figures.
