# Supplementary material for: Can clinicians predict individual patient outcomes in neuroendocrine tumors treated with [177Lu]Lu-DOTATATE?
Source: Oncologist. 2026 Jun 15;31(7):oyag231. doi: 10.1093/oncolo/oyag231 (PMC13302793; doi:10.1093/oncolo/oyag231)

Hierarchical cluster analysis of covariates based on pairwise Hoeffding's D statistics for similarity

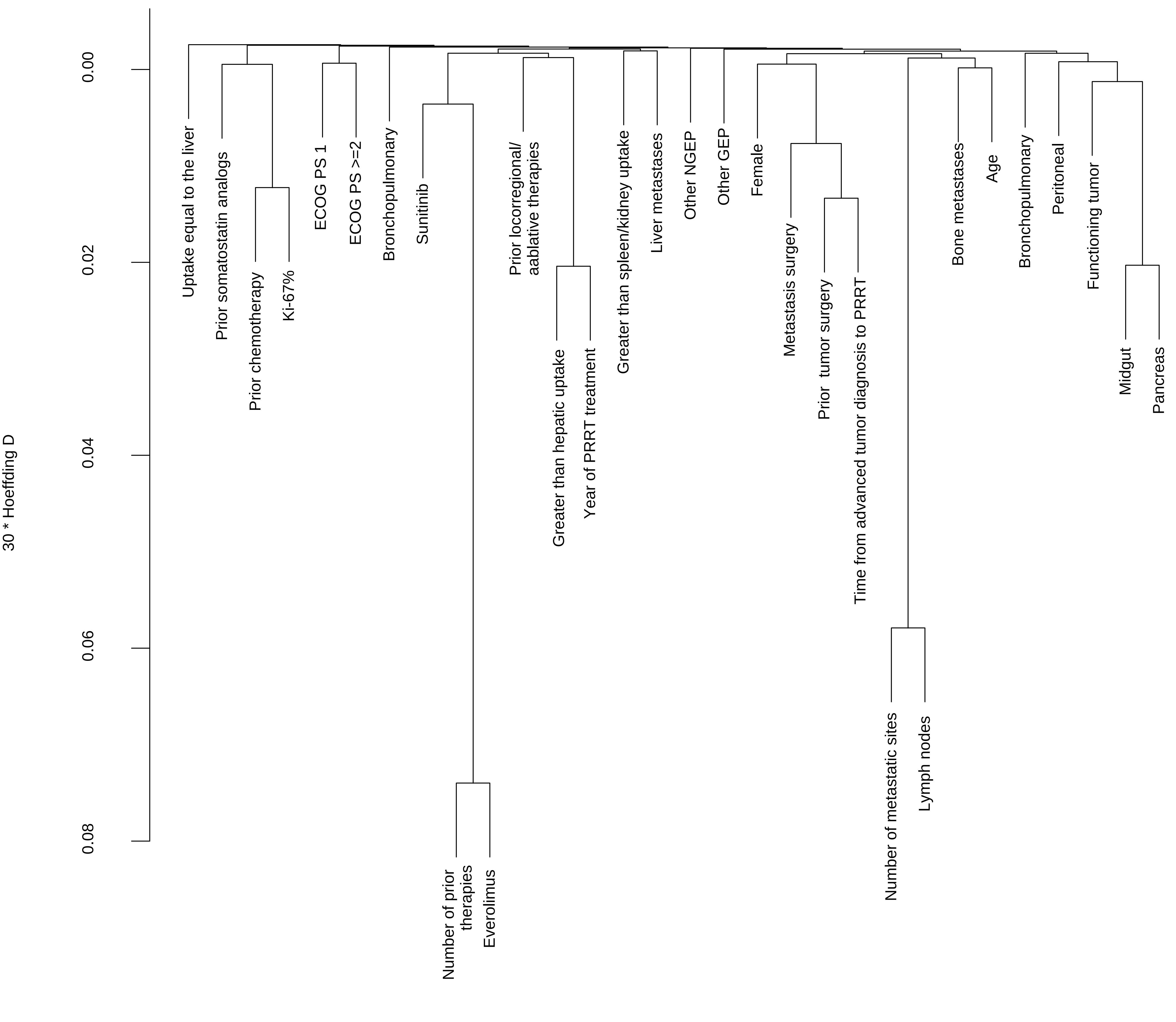

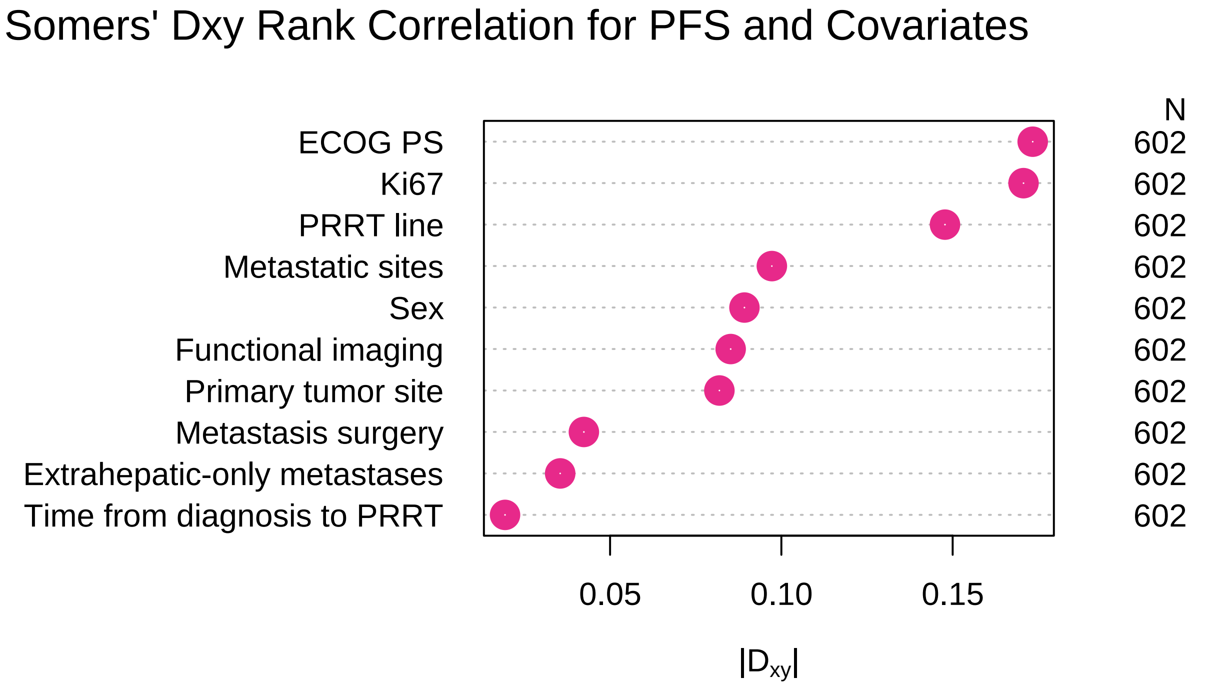

Somers' Dxy Rank Correlation for PFS and Covariates
N
ECOG PS
Ki67
PRRT line
Metastatic sites
Sex
Functional imaging
Primary tumor site
Metastasis surgery
Extrahepatic-only metastases
Time from diagnosis to PRRT
602
602
602
602
602
602
602
602
602
602
0.05
0.10
0.15
|Dxy|

PFS Model: Weibull Distribution

Variable	Coefficient	Standard Error	Wald Z	P-value
(Intercept)	3.5824	0.3631	9.87	<0.0001
ECOG PS 0	0.2914	0.0937	3.11	0.0019
ECOG PS >=2	-0.5859	0.1794	-3.27	0.0011
Sex (Female)	0.2228	0.0959	2.32	0.0202
Functional Imaging (Higher than spleen and kidney)	0.6066	0.2587	2.34	0.0190
Functional Imaging (Higher than liver uptake)	0.436	0.2329	1.87	0.0612
Functional Imaging (PET)	0.5084	0.2385	2.13	0.0330
Primary Tumor Site (Midgut)	0.1285	0.2648	0.49	0.6275
Primary Tumor Site (Other GEP)	0.171	0.2877	0.59	0.5523
Primary Tumor Site (Other NGEP)	0.0991	0.2916	0.34	0.7339
Primary Tumor Site (Pancreas)	-0.1656	0.267	-0.62	0.5351
Primary Tumor Site (Lung)	-0.2129	0.2789	-0.76	0.4452
Number of Metastatic Sites	-0.1202	0.0425	-2.83	0.0047
Ki67	-0.0165	0.0038	-4.36	<0.0001
Spline Component 1: Time from advanced tumor diagnosis to PRRT (months)	-0.0069	0.0044	-1.58	0.1153
Spline Component 2: Time from advanced tumor diagnosis to PRRT (months)	0.0144	0.0058	2.49	0.0126
PRRT Line (3)	-0.4587	0.12	-3.82	0.0001
PRRT Line (4)	-0.6945	0.1247	-5.57	<0.0001

Surgical resection of metastases	0.2805	0.1195	2.35	0.0189
Liver Metastases	0.3363	0.1365	2.46	0.0138

OS Model: Weibull Distribution

Variable	Coefficient	Standard Error	Wald Z	P-value
(Intercept)	4.5846	0.4904	9.35	<0.0001
ECOG PS 0	0.4366	0.1264	3.46	0.0005
ECOG PS >=2	-0.8578	0.1883	-4.56	<0.0001
Sex (Female)	0.1668	0.1231	1.36	0.1754
Functional Imaging (Higher than spleen and kidney)	0.059	0.3612	0.16	0.8702
Functional Imaging (Higher than liver uptake)	0.0068	0.3367	0.02	0.9838
Functional Imaging (PET)	0.364	0.3445	1.06	0.2907
Primary Tumor Site (Midgut)	0.0765	0.3297	0.23	0.8166
Primary Tumor Site (Other GEP)	0.4845	0.3779	1.28	0.1999
Primary Tumor Site (Other NGEP)	-0.006	0.353	-0.02	0.9864
Primary Tumor Site (Pancreas)	-0.0941	0.3303	-0.28	0.7757
Primary Tumor Site (Lung)	-0.1446	0.3456	-0.42	0.6756
Number of Metastatic Sites	-0.1452	0.0536	-2.71	0.0068
Ki67	-0.019	0.0044	-4.3	<0.0001
Spline Component 1: Time from advanced tumor diagnosis to PRRT (months)	-0.002	0.0056	-0.37	0.7145
Spline Component 2: Time from	0.0101	0.0074	1.36	0.1748

advanced tumor diagnosis to PRRT (months)				
PRRT Line (3)	-0.5118	0.1659	-3.08	0.0020
PRRT Line (4)	-0.7498	0.1709	-4.39	<0.0001
Surgical resection of metastases	0.2513	0.1587	1.58	0.1133
Liver Metastases	0.2361	0.1801	1.31	0.1897

Calibration plots for the OS endpoint

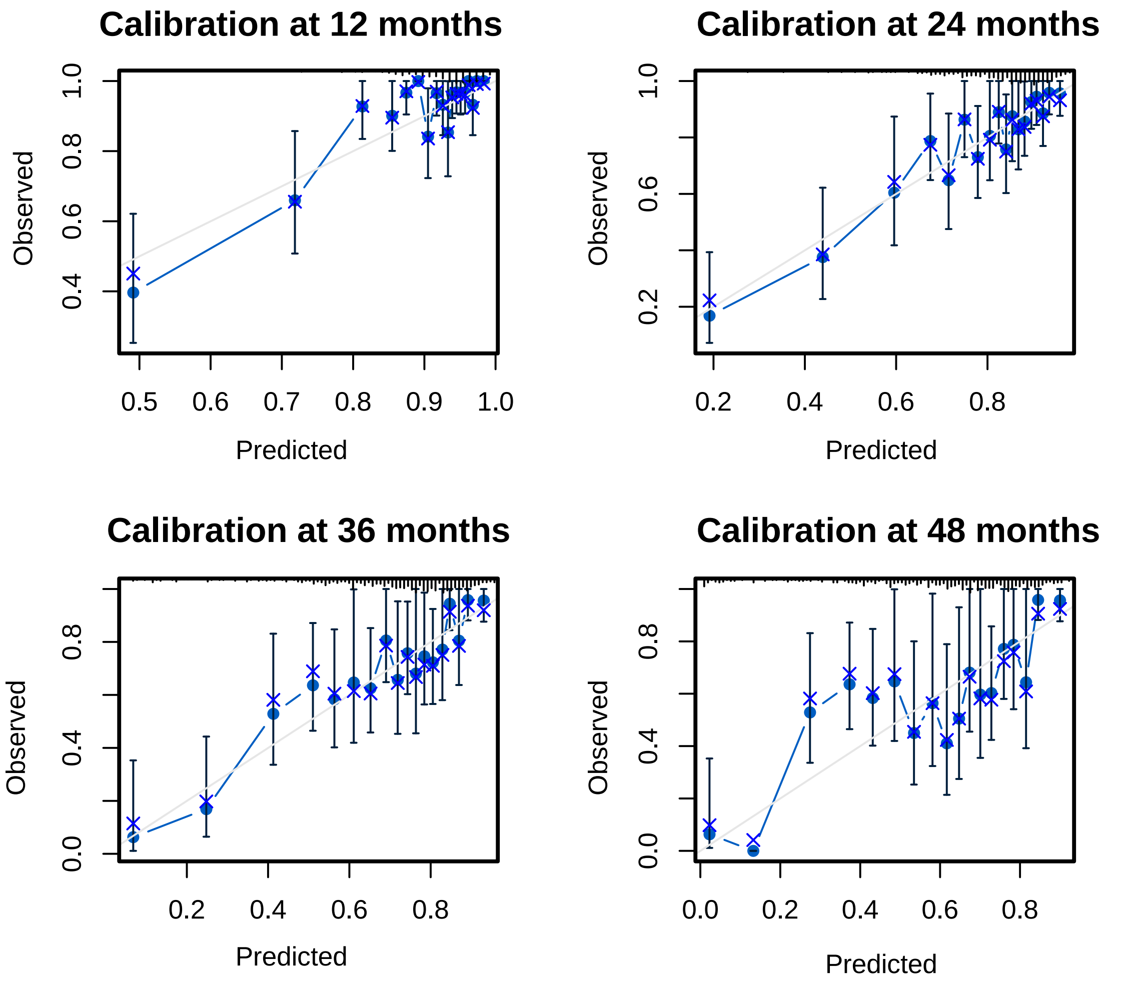

Supplement: oyag231_Supplementary_Data [file oyag231_supplementary_data.zip › Supplementary Appendix.pdf]
