# Supplementary material for: Can clinicians predict individual patient outcomes in neuroendocrine tumors treated with [177Lu]Lu-DOTATATE?
Source: Oncologist. 2026 Jun 15;31(7):oyag231. doi: 10.1093/oncolo/oyag231 (PMC13302793; doi:10.1093/oncolo/oyag231)
Supplement: oyag231_Supplementary_Data [file oyag231_supplementary_data.zip › Supplementary Figure 1.docx]

# **Supplementary Figure S1. Illustrative example: use of the NEPTUNE web-based calculator**

To facilitate clinical uptake, the NEPTUNE score is accessible as an interactive web-based calculator at https://www.prognostictools.es/clinical-calculators/neptune. The figure below shows a worked example for a representative patient profile.


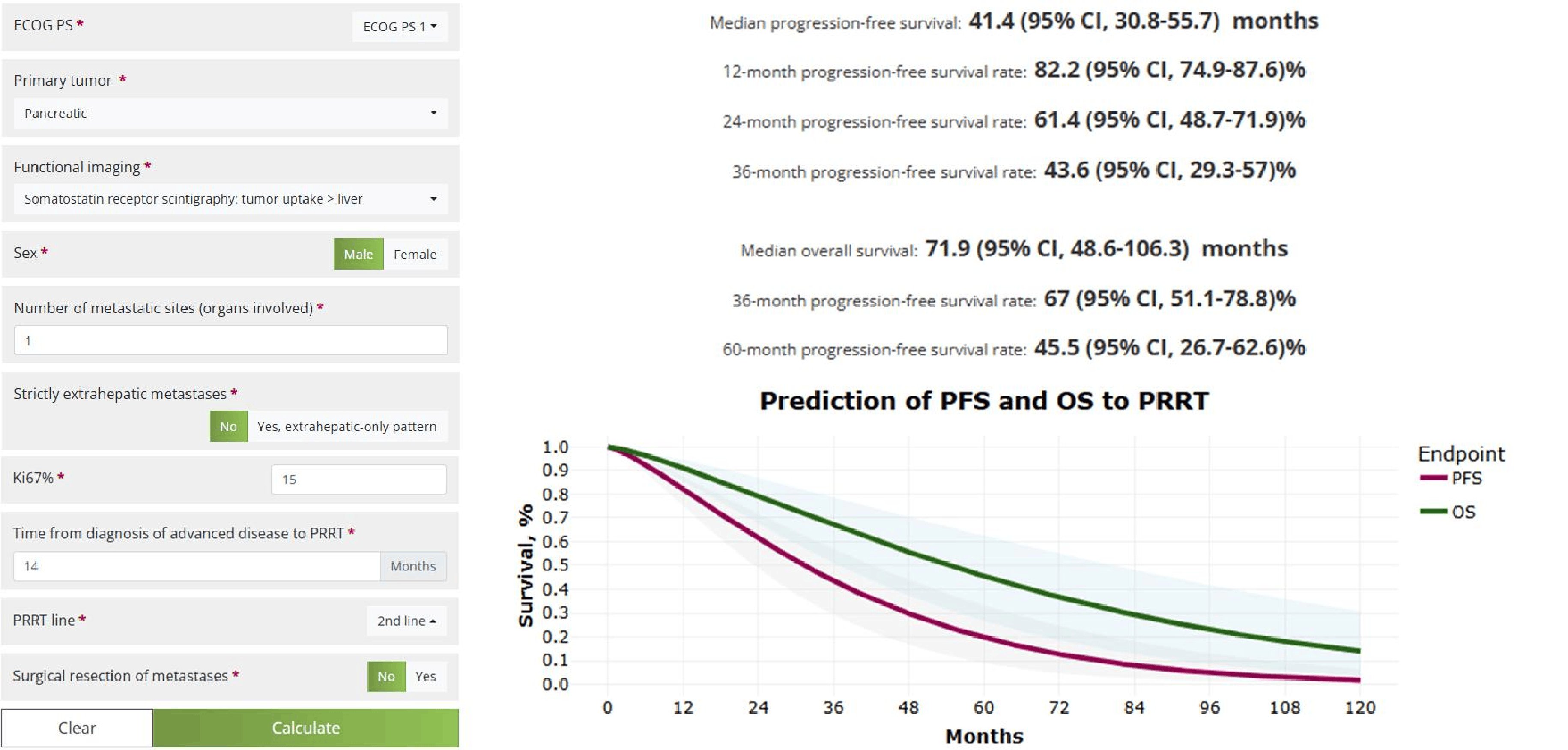


*Worked example. A male patient with a pancreatic NET (Ki-67 15%), ECOG PS 1, one metastatic site, non–extrahepatic-only pattern, Krenning grade > liver uptake, treated with second-line PRRT 14 months after the diagnosis of advanced disease, and no prior metastasectomy. The calculator returns a predicted median PFS of 41.4 months (95% CI, 30.8–55.7), 12-, 24- and 36-month PFS rates of 82.2%, 61.4% and 43.6% respectively, and a predicted median OS of 71.9 months (95% CI, 48.6–106.3). Predicted PFS and OS curves (right panel) are shown with 95% confidence bands. Abbreviations: CI, confidence interval; ECOG PS, Eastern Cooperative Oncology Group performance status; NET, neuroendocrine tumor; OS, overall survival; PFS, progression-free survival; PRRT, peptide receptor radionuclide therapy.*
