# Supplementary material for: Can clinicians predict individual patient outcomes in neuroendocrine tumors treated with [177Lu]Lu-DOTATATE?
Source: Oncologist. 2026 Jun 15;31(7):oyag231. doi: 10.1093/oncolo/oyag231 (PMC13302793; doi:10.1093/oncolo/oyag231)
Supplement: oyag231_Supplementary_Data [file oyag231_supplementary_data.zip › Supplementary Table S1.docx]

# **Supplementary Table S1. Functional imaging categories and mapping into the NEPTUNE model**

Functional imaging findings were harmonised across modalities to ensure consistency in their incorporation into the model. Krenning-scale categories from somatostatin receptor scintigraphy were treated as ordinal levels of increasing SSTR expression; [68Ga]Ga-DOTATOC PET examinations (approximately one third of the cohort) were handled as a separate category because the visual scoring scheme is not directly superimposable on the Krenning scale. Quantitative PET parameters (e.g., SUVmax, total tumor volume) were not included because of high missingness (>65%; see Table S1).

| **Imaging modality** | **Category** | **Ordinal level** | **Operational definition** |
| --- | --- | --- | --- |
| SSTR scintigraphy | Krenning grade 2 | 1 (lowest) | Uptake comparable to liver parenchyma |
| SSTR scintigraphy | Krenning grade 3 | 2 | Uptake greater than liver |
| SSTR scintigraphy | Krenning grade 4 | 3 (highest) | Uptake greater than spleen or kidney |
| [68Ga]Ga-DOTATOC PET | SSTR-positive lesions | Separate PET category | Lesions visually above background (physiological uptake) |

*Abbreviations: PET, positron emission tomography; SSTR, somatostatin receptor; SUV, standardised uptake value.*
