# Supplementary material for: Can clinicians predict individual patient outcomes in neuroendocrine tumors treated with [177Lu]Lu-DOTATATE?
Source: Oncologist. 2026 Jun 15;31(7):oyag231. doi: 10.1093/oncolo/oyag231 (PMC13302793; doi:10.1093/oncolo/oyag231)
Supplement: oyag231_Supplementary_Data [file oyag231_supplementary_data.zip › Supplementary Table S2.docx]

# **Supplementary Table S2. Missingness of candidate predictors and handling strategy**

Missingness percentages are reported for all candidate predictors considered during model development. Variables with more than 20% missing data were excluded a priori, consistent with the recommendation that multiple imputation becomes unreliable beyond this threshold (Harrell, Regression Modeling Strategies, 2nd ed.). Variables with less than 20% missingness were imputed using predictive mean matching (PMM) via the aregImpute function (Hmisc package), with 10 imputations.

| **Candidate predictor** | **Missingness (%)** | **< 20% threshold?** | **Included in model** | **Handling** |
| --- | --- | --- | --- | --- |
| ECOG performance status | 3.4 | Yes | Yes | Multiple imputation (PMM) |
| Sex | 0 | Yes | Yes | Complete data |
| Primary tumor site | 0 | Yes | Yes | Complete data |
| Ki-67 proliferation index | 6.2 | Yes | Yes | Multiple imputation (PMM) |
| Number of metastatic sites | 1.1 | Yes | Yes | Multiple imputation (PMM) |
| Liver metastases (yes/no) | 1.1 | Yes | Yes | Multiple imputation (PMM) |
| Extrahepatic-only pattern | 2.3 | Yes | Yes | Multiple imputation (PMM) |
| Krenning grade / PET category | 4.8 | Yes | Yes | Multiple imputation (PMM) |
| PRRT line | 0.5 | Yes | Yes | Multiple imputation (PMM) |
| Time from advanced diagnosis to PRRT | 0.8 | Yes | Yes | Multiple imputation (PMM) |
| Prior surgical resection of metastases | 6.5 | Yes | Yes | Multiple imputation (PMM) |
| Age | 0 | Yes | Auxiliary* | Complete data |
| Year of PRRT initiation | 0 | Yes | Auxiliary* | Complete data |
| Chromogranin A (baseline) | 42.7 | No | No | Excluded a priori |
| Symptom burden (structured) | 38.5 | No | No | Excluded a priori |
| 5-HIAA (urinary) | 61.4 | No | No | Excluded a priori |
| NSE | 57.2 | No | No | Excluded a priori |
| SUVmax on [68Ga]Ga-DOTATOC PET | 66.9 | No | No | Excluded a priori |
| Ga-PET total tumor volume | 72.1 | No | No | Excluded a priori |
| Baseline hemoglobin | 14.2 | Yes | No† | Not retained (redundancy) |
| Baseline creatinine | 12.8 | Yes | No† | Not retained (redundancy) |

*Abbreviations: 5-HIAA, 5-hydroxyindoleacetic acid; CCA, complete-case analysis; GEP, gastroenteropancreatic; NSE, neuron-specific enolase; PET, positron emission tomography; PMM, predictive mean matching; PRRT, peptide receptor radionuclide therapy; SUV, standardised uptake value; TR, time ratio.*

** Age and year of PRRT initiation were included in the imputation model as auxiliary variables but were not retained as predictors in the final NEPTUNE score. † Laboratory variables with <20% missingness were screened but were not retained after redundancy analysis (Hoeffding's D, variance-inflation factors). Planned inclusion in future model iterations: circulating biomarkers (chromogranin A, NETest), quantitative PET parameters (SUVmax, total tumor volume), and structured symptom burden are envisaged as candidate predictors for a subsequent update of the score, once prospective capture improves completeness.*
