# Supplementary material for: Can clinicians predict individual patient outcomes in neuroendocrine tumors treated with [177Lu]Lu-DOTATATE?
Source: Oncologist. 2026 Jun 15;31(7):oyag231. doi: 10.1093/oncolo/oyag231 (PMC13302793; doi:10.1093/oncolo/oyag231)
Supplement: oyag231_Supplementary_Data [file oyag231_supplementary_data.zip › Supplementary Table S3.docx]

# **Supplementary Table S3. Sensitivity analysis: Multiple Imputation vs Complete-Case Analysis**

To assess the influence of the imputation strategy on the results, the Weibull accelerated failure-time model was refit on the complete-case subset (n = 560; 86.6% of the full sample, 87 observations dropped) and compared with the primary multiple-imputation (MI) fit (n = 647). Coefficients are expressed as time ratios (TR = exp(β)); values >1 indicate a longer expected PFS, values <1 a shorter PFS. Internal validation (C-index, calibration slope) was repeated for each fit using 200 bootstrap resamples.

**S3a. Coefficient comparison**

| **Parameter** | **TR MI (95% CI)** | **p MI** | **TR CCA (95% CI)** | **p CCA** |
| --- | --- | --- | --- | --- |
| ECOG PS 0 (vs 1) | 1.34 (1.11–1.61) | 0.002 | 1.30 (1.08–1.57) | 0.007 |
| ECOG PS ≥2 (vs 1) | 0.56 (0.39–0.79) | 0.001 | 0.49 (0.34–0.70) | <0.001 |
| Female sex (vs male) | 1.25 (1.04–1.51) | 0.020 | 1.27 (1.04–1.54) | 0.018 |
| Krenning: > spleen/kidney | 1.83 (1.10–3.05) | 0.019 | 2.20 (1.30–3.72) | 0.003 |
| Krenning: > hepatic uptake | 1.55 (0.98–2.44) | 0.061 | 1.61 (1.01–2.57) | 0.045 |
| PET (positive) | 1.66 (1.04–2.65) | 0.033 | 1.76 (1.09–2.83) | 0.021 |
| Site: midgut | 1.14 (0.68–1.91) | 0.628 | 2.11 (1.13–3.96) | 0.020 |
| Site: other GEP | 1.19 (0.68–2.09) | 0.552 | 2.29 (1.18–4.45) | 0.015 |
| Site: other non-GEP | 1.10 (0.62–1.96) | 0.734 | 2.20 (1.10–4.41) | 0.026 |
| Site: pancreas | 0.85 (0.50–1.43) | 0.535 | 1.50 (0.80–2.82) | 0.207 |
| Site: bronchopulmonary | 0.81 (0.47–1.40) | 0.445 | 1.46 (0.76–2.77) | 0.254 |
| Tumor burden (per 1 site) | 0.89 (0.82–0.96) | 0.005 | 0.89 (0.82–0.97) | 0.010 |
| Ki-67 (per unit, continuous) | 0.98 (0.98–0.99) | <0.001 | 0.98 (0.98–0.99) | <0.001 |
| PRRT line 3 (vs 2) | 0.63 (0.50–0.80) | <0.001 | 0.70 (0.55–0.89) | 0.004 |
| PRRT line ≥4 (vs 2) | 0.50 (0.39–0.64) | <0.001 | 0.61 (0.47–0.79) | <0.001 |
| Prior metastasectomy | 1.32 (1.05–1.67) | 0.019 | 1.23 (0.97–1.57) | 0.094 |
| Liver metastases | 1.40 (1.07–1.83) | 0.014 | 1.33 (0.99–1.78) | 0.058 |

**S3b. Internal validation — discrimination and calibration**

| **Performance metric** | **Multiple Imputation (n = 647)** | **Complete-Case (n = 560)** |
| --- | --- | --- |
| C-index (apparent) | 0.720 | 0.711 |
| C-index (bootstrap-corrected) | 0.702 | 0.690 |
| Optimism (Dxy) | 0.035 | 0.041 |
| Calibration slope (apparent) | 1.000 | 1.000 |
| Calibration slope (bootstrap-corrected) | 0.905 | 0.888 |

*The MI and CCA fits yielded closely concordant coefficients for the dominant predictors (ECOG, Ki-67, PRRT line, tumor burden, sex, Krenning grade) and comparable bootstrap-corrected discrimination (C-index 0.702 vs 0.690) and calibration slope (0.905 vs 0.888), supporting the robustness of the NEPTUNE model to the handling of missing data. Minor differences were observed for the primary-site coefficients, as expected given the reduced stratum sizes in the complete-case subset. AIC/BIC are not directly comparable between fits because they are computed on different sample sizes. Abbreviations: AIC, Akaike information criterion; BIC, Bayesian information criterion; CCA, complete-case analysis; CI, confidence interval; MI, multiple imputation; PS, performance status; TR, time ratio.*
